# The concept of marketing in the public-private partnership in the medical system in Romania

**Published:** 2014

**Authors:** VL Purcărea, BI Coculescu, EC Coculescu

**Affiliations:** * ”Carol Davila” University of Medicine and Pharmacy, Bucharest, Romania; ** Faculty of Medicine, “Titu Maiorescu” University, Bucharest, Romania; *** Committee of Public Health of the Senate, Bucharest, Romania

**Keywords:** public-private partnership, Romanian healthcare system, healthcare marketing

## Abstract

During the transition period, the Romanian medical system, subject (like other areas) to a process of reform, had to go through a difficult path, not without obstacles (malpractice, underfunding, embezzlement scandals in the media etc.). Consequently, Romania has faced (and unfortunately still is still facing) the massive exodus of health professionals to countries where they can benefit from better working conditions and payment, and those who suffer from health care crisis are the patients. Reform means “the people in the system for the people”, which requires a change of mindset within the medical staff, especially in the continuous professional development. However, to talk about the quality of the medical act requires that all those involved in the medical system should create appropriate conditions – i.e. advanced technical equipment and appropriate salaries. In addition, as underfunding is the main cause of failure in the health system, that management remains the only tool that can lead to the appropriate use of the existing resources and the quality of health services. Therefore, the idea of public-private partnership – which occurred as a challenge, especially after the EU accession - can be considered a solution designed to improve the quality and cost of health services. In other words, the cooperation of the private sector with the public authority means increasing the rigor of the medical equipment performance, fostering professional competition, and an increased attention to the patient, in a word: performance.

Currently, more and more frequently, the management has studied to identify opportunities for innovation in health care services in an attempt to bring together patients and practitioners in the field by resorting to the identification of the ways they can receive health care services promptly, fairly and efficiently. Therefore, a clear and responsible design in the spirit of ethics and medical ethic will help the marketing manager solve many of the complex issues raised by the marketing activity in the field of health care.

## Introduction

Even if the Romanian medical system is in the process of reform, by benefiting from a package of laws aimed at upgrading, we allow ourselves to make some considerations concerning the “continuous need for health reform”. Why? Precisely because, in our opinion, more needs to be done in this area, which is so sensitive equally for patients and for those working in the medical system. We would like to refer to the latter in particular, on the assumption that they themselves must be the beneficiaries of the reforms in the system.

## Discussion

It is known that the Romanian medical system is in the process of a cultural change, which takes time and patience, and perseverance, despite the unpredictability and resistance that it faces inevitably. This painful period of transition is a commonly known admitted fact, Romanian medicine - like other areas otherwise - has traveled a windy road, often being hard for it to overcome obstacles (malpractice, unnecessary medical scandals in the media, embezzlement, underfunding, etc.) [**1**].

As it is known, the success of a reform depends, largely, on how it is understood to be subsequently treated and accepted. According to a recent sociological study on the “Quality of working life and trend of migration of health care personnel” [**2**], only 6.5 percent of the medical staff has a good opinion on the functioning of the system, while 76.3% of the respondents consider that the current working conditions do not provide an adequate framework for the provision of a quality medical service. In this context, we consider it necessary, more than ever - taking into account the fact that Romania became a full member of the EU - to pay attention to the improvement of the current poor infrastructure (hospitals, insufficient obsolete equipment). To all these aspects, the underfunding of the system, which had a negative impact on the quality of medical care, and the living standards of the medical staff were added. Consequently, we are facing a mass exodus of health professionals to countries where they benefit from much better working conditions and payment. Finally yet importantly, those who were directly affected were the patients.

On the other hand, we must recognize and raise a warning: while leaving to work abroad, an increasingly larger number of those working in the medical system will inevitably lead to a shortage of staff in the hospitals in Romania, a phenomenon that is already being felt [**1**].

Moreover, other paradoxes need to be brought forward too: low wages, not differentiated according to the risk and performance, uniformity of incomes, significantly differentiated malpractice (equal pay, specialist surgery pay double amount compared to other surgeries), lack of insurance risk, officials who undergo surgeries abroad, “ordinary people” who undergo surgeries abroad, etc. A problem also caused by the underfunding is the shortage of stocks of drugs and supplies in Romanian hospitals for disaster situations. Finally, the attention should be drawn to another aspect, that of the need to change attitudes about the medical staff, thus continuing professional development because, unfortunately, according to the same survey, only 27 percent of the respondents declared their concern for the information presented in their professional field.

Also concerning the change of mentality, we emphasized that the reform will be complete if we, the potential patients, will look differently at people in the medical system, because ultimately “the reform of the system is made by people for people”. All those working in the operating theaters, laboratories and medical practices in Internal Medicine and Imaging saving lives and healing a lot of suffering, should at least be provided with advanced technical features, and must have the corresponding salary income.

Because only this medical formula can really bring high quality to the medical act.

After joining the European Union, the Romanian medical system in general, and health services management faced new challenges. It is widely recognized that health systems need a private dimension. In this respect, the public-private partnership is a solution, especially in situations in which the state has little money to start a project. Perpetual funding crisis requires finding solutions to improve the quality of the medical care in general. In fact, health is a strategic resource of every nation and must become cost effective. Therefore, confinement must demonstrate that health is not only costs but also investment, which can become profitable. In other words, the presence of a public-private partnership can lead to a rapidly increased quality and to efficient health services. However, this type of partnership can provide an optimal alternative for the private sector to cooperate with public authorities providing private capital and the necessary expertise to ensure public health services.

The advantages of a public-private partnership in health are rigor of care, provision of equipment performance, attention to patients and, last but not least, stimulation of professional competition, hence leading to performance.

Recent studies showed that the health indicators in Romania have seen a steady improvement, but it is necessary to continue the reform process so that we can reach the standards of EU countries in health.

In this respect, the implementation of a public-private partnership can be a driver of the health system development. Currently, in Romania, in Bucharest and the other major cities, a number of hospitals and clinics already operate successfully in PPP mode. We hope that this system (public-private) will extend both to the benefit of patients and to the stimulation of professional performance. This is especially since quality is a problem for most public medical institutions, while providing quality benefits are obvious and without exception recognized both by consumers and by suppliers.

To meet shortfalls caused by the economic crisis, the Romanian medical system needed an objective analysis of the quality of medical care as a whole, the entire package of health services and accountable joint efforts to identify the system problems and, especially, a firm action without compromising the resolution, regardless of any limitations or emotional picture. In addition, last but not least, the judicious use of available resources is also important.

Budgetary restrictions require the improvement of health care quality in the Romanian medical system because its absence leads to a duplication of services, and thus to an increase in spending. Unfortunately, this is still observed in the health services, even if, from the point of view of the technical facilities, great progress has been made in the last decade of the last century, however, quality standards are not met everywhere in Romania and there is a waste of resources through duplication of investigations, analyses, etc.

To this end, the audit work and reporting medical health system (e.g., DRG’s for hospitals) is a mechanism to be supported at all times to avoid the duplication of services, to pay providers (particularly hospitals) according to the medical activities actually carried out and to respect the principle of “money follows the patient”.

The insufficient funding is the most common explanation given for the lack of quality health services and it is indeed a problem. Given this circumstance, the management remains the only tool that can lead to an appropriate use of the existing resources and the quality of health services. One of the new concepts that began to work in this field is Total Quality Management (TQM), a general organizational model involving the planning and implementation of a continuous quality improvement process that exceeds customer expectations. This model assumes that 90% of the problems are process issues, not staff issues. Total Quality Management is not a new concept, but has not been fully developed in services. It is based on the rule of “doing things right the first time” and prevention. It is a preventive strategy that eliminates double work, remedial efforts at the last minute and it is based on planning, coordination and control [**3**].

As an economic principle, the less resources are used to achieve the expected results, the more effective the supplier is. Nevertheless, the introduction of new medical technologies, many of them more notable but more expensive, requiring a reassessment of the way resources are used with the suppliers to produce the expected results, the evaluation based on cost-effectiveness/ analysis (or per patient), are important elements.

Finally yet importantly, the medical laboratory paraclinical services are the tools of the marketing strategy of any medical organization without which the needs and motivations of the beneficiaries of these services (patients) could not be satisfied. In essence, the entire marketing philosophy is based on the needs and wishes of the people as well as the practical solutions to solve them.

Today, the challenge for all those involved in the health care service system is how to answer the five questions of the customer-oriented marketing, the time of first contact with the client / patient/ health service: ask the client what he/ she really wants; focus on what the customer thinks is important; make sure you act on feedback from your client; get feedback on an ongoing basis; create feedback loops throughout your company.

Based on the recommendation that “if you cannot service differently then differentiate yourself by offering more services” [**4**], the more frequent is the recourse to management studies to identify opportunities for innovation in health care services in an attempt to bring together patients and practitioners to identify how that can best meet the latter and, especially, how to take advantage of health care services promptly, fairly and efficiently.

Obviously marketing situations differ, but the essence of successful organizations concerned notifies the importance of strategic alignment of relations between all who have key interests in a medical facility - shareholders, managers, health professionals, patients - and the consequences of these interactions to improve medical service quality and thereby the performance and profitability of the institution as such and even the relational landscape as a whole, making patients true brand promoters, thus multiplying marketing efforts.

The success of the healthcare organizations is subject to the accurate knowledge of the expectations of consumers / clients / patients, government requirements and the competitors’ criticisms, and society in general, in terms of social responsibility. Respect for the patients’ rights is one of the most important issues of social responsibility in the protection of the beneficiaries of health services aiming at the achievement of objectives, including performance and reliability of the service, provision of relevant information on the services offered, etc. [**5**].

## Conclusions

Each medical organization and marketing manager must adopt a socially responsible attitude, to look only within the legal framework and implement standards based on long-term health and welfare of patients. In addition, a clear concept and responsible medical ethics and deontology spirit will help the marketing manager solve many of the complex issues raised by the marketing activity in the field of health care.
